# Supplementary material for: Initial Response of Pentaerythritol Tetranitrate (PETN) under the Coupling Effect of Preheating, Shock and Defect via the Molecular Dynamics Simulations with the Multiscale Shock Technique Method
Source: Molecules. 2023 Mar 24;28(7):2911. doi: 10.3390/molecules28072911 (PMC10096352; doi:10.3390/molecules28072911)
Supplement: Supplementary file 1 [file molecules-28-02911-s001.zip › molecules-2256215-supplementary.docx]

Supplementary Information

**Initial response of pentaerythritol tetranitrate (PETN) under the coupling effect of preheating, shock and defect via the molecular dynamics simulations with the Multiscale Shock Technique method**

Yaping Zhang ^a, b^, Tao Wang ^a,^ *, and Yuanhang He ^c,^ *

^a^ Institute of Intelligent Manufacturing Technology, Shenzhen Polytechnic, Shenzhen, 518055, China

^b^ Shenzhen Institutes of Advanced Technology, Chinese Academy of Sciences, Shenzhen, 518055, China

^c^ State Key Laboratory of Explosion Science and Technology, Beijing Institute of Technology, Beijing 100081, China

**S1. The method details of Multiscale Shock Technique.**

In the MSST method, the one-dimensional Euler equation based on a compressible fluid can be expressed as:

| $\frac{d\rho}{dt}+\rho\frac{\partial u}{\partial x}=0$ | (1) |
| --- | --- |
| $\frac{du}{dt}+\tilde{v}\frac{\partial p}{\partial x}=0$ | (2) |
| $\frac{d\tilde{e}}{dt}+p\frac{d\tilde{v}}{\mathrm{dt}}=0$ | (3) |

where $\rho$ is the density, $u$ is the local material velocity, $\tilde{v}$ is the specific volume, *p* is the pressure, and $\tilde{e}$ is the energy per unit mass. Through coordinate transformation, the Hugoniot relation for the shock wave velocity of Vs can be obtained:

| $u-u_{0}=\left( V_{s}-u_{0} \right)\left( 1-\frac{\rho_{0}}{\rho} \right)$ | (4) |
| --- | --- |
| $p-p_{0}=\left( u_{0}-V_{s} \right)^{2}\rho_{0}\left( 1-\frac{\rho_{0}}{\rho} \right)$ | (5) |
| $\tilde{e}-\tilde{e}_{0}=p_{0}\left( \frac{1}{\rho_{0}}-\frac{1}{\rho} \right)+\frac{\left( u_{0}-V_{s} \right)^{2}}{2}\left( 1-\frac{\rho_{0}}{\rho} \right)^{2}$ | (6) |

where the subscript 0 denotes the physical quantity of the material in front of the shock wave and $u$ is the particle velocity.

In molecular dynamics calculations, the MSST technique implements the above Hugoniot relationship by establishing the Hamiltonian equation of motion:

| $\tilde{E}=\tilde{e}\left( \left\{ \dot{\vec{r_{i}}} \right\},\left\{ \vec{r_{i}} \right\} \right)+\frac{1}{2}Q{\dot{\tilde{v}}}^{2}-\frac{v_{s}^{2}}{2}\left( 1-\frac{\tilde{v}}{\tilde{v}_{0}} \right)+p_{0}\left( \tilde{v}-\tilde{v}_{0} \right)$ | (7) |
| --- | --- |

where Q is the class mass parameter in units of mass^2^/crystal length^4^.

**S2. ReaxFF reactive force field.**

ReaxFF is a first-principles-based force field, and the bond strength and bond length calculated by ReaxFF can adjust appropriately in response to variations of the local chemical environment. The ReaxFF is a bond-order potential based from quantum mechanical (QM) calculations, which provides accurate descriptions of complex chemical reactive processes. ReaxFF/lg corrects the London dispersion interaction by adding an extra term using a low-gradient model. The main formula is as follows:

$$\text{E}_{\text{Reax-lg}}\text{= }\text{E}_{\text{Reax}}\text{+ }\text{E}_{\text{lg}}$$

$$\text{E}_{\text{Reax}}\text{=}\text{E}_{\text{bond}}\text{+}\text{E}_{\text{lp}}\text{+}\text{E}_{\text{over}}\text{+}\text{E}_{\text{under}}\text{+}\text{E}_{\text{val}}\text{+}\text{E}_{\text{pen}}\text{+}\text{E}_{\text{coa}}$$

$$\text{ }\text{+}\text{E}_{\text{tors}}\text{+}\text{E}_{\text{conj}}\text{+}\text{E}_{\text{H-bond}}\text{+}\text{E}_{\text{vdWaals}}\text{+}\text{E}_{\text{Coulomb}}$$

$$\text{E}_{\text{lg}}\text{= -}\sum_{\text{ij,i<j}}^{\text{N}} \frac{\text{C}_{\text{lg,ij}}}{\text{r}_{\text{ij}}^{\text{6}}\text{+d}\text{R}_{\text{eij}}^{\text{6}}}$$

Where $E_{Reax}$ is energy evaluated from the previous ReaxFF force field, $E_{lg}$ is the long-range-correction terms using the low-gradient model, *r_ij_* is the distance between atom *i* and atom *j*, $R_{eij}^{6}$ is the equilibrium vdW distance, $C_{lg,ij}$ is the dispersion energy correction parameter.
